# Oil of Gaultheria

**Published:** 1883-09-20

**Authors:** J. H. Logan

**Affiliations:** Georgia


### OIL OF GAULTHERIA.

BY J. H. LOGAN, M. D., OF GEORGIA.

I wish to give to the readers of your Journal the benefit of my experience with the oil of gaultheria in the treatment of acute rheumatism. The patient was a female, 18 years of age. Both wrist joints and the joints of all the fingers was attacked; the pain and swelling were considerable, with high fever. The patient could not move a single joint affected. I put her upon 15 grain doses of salicylic acid every six hours with opiates to relieve the pain. This treatment was kept up three days with no relief, in fact the patient continued to grow worse. The salicylic acid was discontinued and the alkaline treatment substituted; this was pushed to the extent of saturation, still no relief was obtained, all seemed as though my patient could not survive another day without help.

Realizing the little good accomplished by the remedies used, I cast about me for more efficient weapons to fight the enemy with. I noticed in the SOUTHERN MEDICAL RECORD, in the March number, 1882, on page 110, what Mr. P. Casamajor says in regard to the oil of gaultheria, I determined to try it, and at once put my patient upon the following:

℞ Salicylic acid .................................. ʒ ij,
  Oil gaultheria ................................. ʒ j,
  Sul. quinine .................................... grs. xij,
  Carb. magnesia ................................ grs. xv.
M. Make into 12 powders. Sig. One every 4 hours.

The carb. magnesia and oil were first triturated together and the other ingredients added and thoroughly mixed.

After the third dose the pain and fever began to subside, and in two days there was a very perceptable change for the better, the pain, swelling and fever rapidly subsiding, and the patient going on to convalescence. In six days after this treatment was commenced the patient was discharged cured. I am confident the cure was due to the use of the oil of gaultheria, as the salicylic acid had been sufficiently tried.

I hope the readers of your Journal will give the oil a trial and report. I for one (with this remedy in my hands) cannot agree with the distinguished doctor who said, "It takes six weeks and a blanket to cure rheumatism."

